# Periodontal therapeutics: Current host‐modulation agents and future directions

**DOI:** 10.1111/prd.12315

**Published:** 2019-12-18

**Authors:** Lorne M. Golub, Hsi‐Ming Lee

**Affiliations:** ^1^ Department of Oral Biology & Pathology School of Dental Medicine Stony Brook University Stony Brook New York, USA

## Abstract

With the recognition in the 1960s and 1970s of the periodontopathic importance of the microbial biofilm and its specific anaerobic microorganisms, periodontitis was treated as an infectious disease (more recently, as a dysbiosis). Subsequently, in the 1980s, host‐response mechanisms were identified as the mediators of the destruction of the collagen‐rich periodontal tissues (gingiva, periodontal ligament, alveolar bone), and the periodontopathogens were now regarded as the "trigger" of the inflammatory/collagenolytic response that characterizes actively destructive periodontitis. Also at this time a new pharmacologic strategy emerged, entitled "host‐modulation therapy", based on 2 major findings: (1) that the ability of tetracycline antibiotics to inhibit periodontal breakdown was due (in large part) to their previously unrecognized ability to inhibit the host‐derived matrix metalloproteinases (notably, the collagenases, gelatinases, macrophage metalloelastase), and by mechanisms unrelated to the antimicrobial properties of these medications; and (2) that nonsteroidal anti‐inflammatory drugs, such as flurbiprofen, again by nonantimicrobial mechanisms, could reduce the severity of periodontitis (however, the adverse effects of long‐term therapy precluded their development as safe and effective host‐modulatory agents). Additional mechanistic studies resulted in the development of novel nonantimicrobial formulations (Periostat® [now generic] and Oracea®) and compositions of tetracyclines (notably chemically modified tetracycline‐3) as host‐modulator drugs for periodontitis, arthritis, cardiovascular and pulmonary diseases, cancer, and, more recently, for local and systemic bone loss in postmenopausal women. Identification of the cation‐binding active site in the tetraphenolic chemically modified tetracycline molecules drove the development of a new category of matrix metalloproteinase‐inhibitor compounds, with a similar active site, the biphenolic chemically modified curcumins. A lead compound, chemically modified curcumin 2.24, has demonstrated safety and efficacy in vitro, in cell culture, and in vivo in mouse, rat, rabbit, and dog models of disease. In conclusion, novel host‐modulation compounds have shown significant promise as adjuncts to traditional local therapy in the clinical management of periodontal disease; appear to reduce systemic complications of this all‐too‐common "inflammatory/collagenolytic" disease; and Oracea® is now commonly prescribed for inflammatory dermatologic diseases.

## 1 INTRODUCTION

In this article we focus on a pharmacologic strategy for managing patients with chronic inflammatory periodontal disease. This strategy, termed “host‐modulation therapy”, was developed almost 3 decades ago by Golub et al^1^, ^2^. To date, the only host‐modulation therapy used clinically in the USA (approved by the US Food and Drug Administration) and beyond (Canada, Europe) is a nonantibiotic formulation of doxycycline, a member of the tetracycline antibiotics (discussed below). This seemingly counterintuitive approach arose from seminal discovery experiments more than 3 decades ago,^3^, ^4^, ^5^, ^6^, ^7^ which resulted in an initial series of review articles a few years later that proposed the clinical use of this nonantibiotic formulation as a novel, safe, and effective therapeutic strategy as an adjunct to scaling and root planing. 1, 2, 6, 7 This strategy has also been tested in surgical regimens of periodontal therapy.^9^

As reviewed in several publications since, 10, 11, 12, 13, 14, 15, 16 2 major categories of host‐modulation therapy have received the most attention. The first category modulates the host's inflammatory response either by inhibition^18^ or, as described more recently, by resolution.^15^, ^16^, ^17^, ^19^, ^20^, ^21^ The second category (the main focus of this chapter) modulates the host's pathologic collagenolytic response in the soft tissues (gingiva and periodontal ligament), as well as the alveolar bone. It should be stressed that collagens in periodontal tissues, comprised mostly of type I but also other collagens, such as type III, are the major structural proteins of all of these soft and calcified tissues. In fact, this ubiquitous fibrous protein comprises over 90% of the organic matrix of the calcified periodontal tissues, the bone, and the cementum, and about 60% of the gingiva and periodontal ligament.^1^, ^2^

Regarding the first category of host‐modulation therapies,2 approaches have been intensively investigated. The earliest studies involved nonsteroidal anti‐inflammatory drugs, but this strategy has been rejected. In brief, the nonsteroidal anti‐inflammatory drug that received the most attention in animal studies, and then in clinical trials, was flurbiprofen. Similar to other nonsteroidal anti‐inflammatory drugs, flurbiprofen suppresses the host's inflammatory response, including its well‐known mediators (eg, prostanoids, cytokines), but also inhibits osteoclast activity and bone resorption.^18^ However, because of significant adverse events in long‐term clinical trials testing nonsteroidal anti‐inflammatory drug efficacy in periodontal patients, including a rebound effect of accelerated alveolar bone loss after cessation of this drug,^22^ these compounds have not been approved for clinical use as a host modulator by governmental regulatory agencies in any country.

In contrast to nonsteroidal anti‐inflammatory drugs, there is great interest in a new category of host‐modulation therapies to regulate inflammation. These novel compounds, the resolvins, do not suppress acute inflammation, which is essential to combat infection and to promote optimal wound healing, but they do prevent its prolongation. These compounds include derivatives of omega‐3 fatty acids, docosahexanoic acid, eicosapentanoic acid, as well as lipoxins derived from arachidonic acid.^19^, ^20^, ^21^ These host‐modulation therapies are not discussed herein as they are detailed in another article in this volume.

The primary focus of this article is the second category of host‐modulation therapies (see above), which modulate the host's pathologic collagenolytic response during periodontitis and other diseases.^1^, ^2^, ^23^ One group of these drugs is based on novel nonantibiotic modifications of tetracyclines (notably doxycycline). The formulations include 2 Food and Drug Administration (and Canada and Europe)‐approved medications for the management of chronic inflammatory periodontal disease and chronic inflammatory skin disease, and both are administered systemically by the oral route. They include Periostat^®^, now generic, and Oracea^®^, the latter widely prescribed by dermatologists and some periodontists.^2^, ^13^, ^24^ The only nonantimicrobial compositions of tetracyclines, i.e., chemically‐modified tetracycline‐3: CMT‐3 [6‐demethyl 6‐deoxy 4‐de‐dimethylamino tetracycline], also known as COL‐3, that has been tested in preliminary clinical trials on patients, are those with periodontal disease 25 and in patients with cancer (Kaposi's sarcoma).^26^ However, none of the chemically modified tetracyclines has been government approved for treating patients in any country.

The second group of novel anticollagenolytic compounds has not yet been approved for clinical use in humans, but has demonstrated safety and efficacy in various animal (mouse, rat, rabbit, dog) models of periodontal disease and relevant medical diseases, such as arthritis, diabetes, and cancer. These compounds are novel chemical modifications of curcumin, the key ingredient of the natural spice, turmeric. In this review, we focus on the most promising of this category, chemically modified curcumin‐2.24, a triketonic phenylaminocarbonyl curcumin (similar in structure to natural diketonic curcumin but different from other diketonics, like the tetracyclines), and its mechanisms of action as a pleiotropic matrix metalloproteinase inhibitor for inflammatory/collagenolytic diseases.^27^, ^28^, ^29^, ^30^, ^31^, ^32^

To complete this chapter, other host‐modulation strategies will be briefly addressed, including the sirtuins and resveratrol.

## 2 CURRENT CLINICAL HOST‐MODULATION AGENTS: DISCOVERY AND THERAPEUTIC RATIONALE

Since the initial discovery experiments of Golub et al,^5^, ^6^ over 40 years ago (see below), the earliest review articles detailing the previously unrecognized multiple mechanisms of action of tetracycline antibiotics, as host modulators, were those published by the same group.^1^, ^2^ These research efforts focused on: (1) the ability of different formulations of subantimicrobial‐dose doxycycline and compositions (eg, the chemically modified tetracyclines) as nonantimicrobial tetracyclines, to inhibit the pathologic breakdown of collagen‐rich tissues, including the resorption of bone; and (2) to explain their mechanisms of action, which are outlined in Table 1 and are discussed briefly below. More than 10 years later, the field had expanded so dramatically in both dentistry and medicine that 2 issues of *Pharmacological Research* 33, 34 were published following a number of related scientific symposia (eg, the New York Academy of Science, The Gordon Research Conferences, etc.) dedicated to the multiple clinical uses of these tetracycline‐based host modulators (mostly doxycycline) for a number of “inflammatory/collagenolytic” diseases, including (but not limited to) periodontitis, arthritis, dermatologic diseases, cardiovascular and lung diseases, and cancer. In addition, the discovery of tetracyclines as host modulators for various medical as well as dental diseases was highlighted in 2 editorials published in *JAMA*.^35^, ^36^

**Table 1:** Nonantibiotic tetracyclines as host modulators inhibit connective tissue breakdown: pleiotropic mechanisms of action^a^

A. Extracellular mechanisms Direct inhibition of activated matrix metalloproteinases in connective tissues, dependent on Zn^++^ and Ca^++^ binding by nonantibiotic tetracyclines Inhibition of promatrix metalloproteinase activation by reactive oxygen species scavenging, independent of cation binding by nonantibiotic tetracyclines Inactivation (by partial proteolysis) of promatrix metalloproteinases, dependent on the binding of cations by nonantibiotic tetracyclines Indirect inhibition of serine proteinases (eg, elastase) by preventing the matrix metalloproteinase‐mediated breakdown of serum alpha_1_‐proteinase inhibitor (ie, alpha1‐PI, also known as alpha1‐antitrypsin)
B. Cellular mechanisms Decreased expression of inflammatory cytokines, nitric oxide, and phospholipase A_2_, thus suppressing promatrix metalloproteinase expression
C. Proanabolic effects “Upregulated” collagen synthesis, osteoblast activity, and bone formation

a Modified from Golub et al.^2^

As background, and of relevance to the drug‐discovery experiments (for more detail, see Golub et al^1^, ^2^), a series of studies was designed to elucidate abnormalities in collagen structure, synthesis, crosslinking/maturation, and degradation, as a complication of experimental diabetes. These experiments led to the discovery that this hyperglycemic state upregulated the synthesis and secretion of the only host‐derived neutral proteinases, the collagenases (ie, matrix metalloproteinase‐1, ‐8, and ‐13), capable of degrading the triple‐helical collagen molecule under physiologic conditions of pH and temperature.^4^, ^37^ Consistent with this observation, diabetes was also found to result in accelerated aging of collagen in connective tissues (including gingiva, skin, bone matrix), characterized by a reduction in the ratio of newly synthesized uncrosslinked (soluble) collagen to older highly crosslinked (insoluble) collagen. The production of excess collagenase provided a mechanism (in addition to decreased procollagen synthesis and excessive lysyl oxidase/crosslinking enzyme activity), the newly synthesized collagen is much more susceptible to collagenolytic attack than the older “leather‐like” highly crosslinked collagen. Also consistent with the high level of diabetes‐induced pathologically‐excessive matrix metalloproteinase (collagenolytic) activity was the excessive urinary excretion of hydroxyproline (an amino acid unique to collagen molecules), compared with that in normal glycemic rats, and the accelerated breakdown of the collagen‐rich periodontal (and other) tissues often seen in patients with diabetes.^4^, ^37^ As discussed below, these observations of accelerated breakdown of recently synthesized collagen, mediated by excessive matrix metalloproteinase activity in rats with diabetes, has been translated into clinical applications. In this regard, Frankwich et al^38^ reported that the treatment of obese patients with type 2 diabetes, with the doxycycline‐based matrix metalloproteinase inhibitor detailed below, “resulted in decreased inflammation and improved insulin sensitivity.” The mechanism proposed was that pathologically elevated matrix metalloproteinase activity during diabetes, which is known to degrade the insulin receptor (thus impairing glucose metabolism), was significantly reduced by a 3‐month treatment with doxycycline, a proven matrix metalloproteinase inhibitor. Earlier, Lauhio et al 39 had shown that matrix metalloproteinase‐8 is effective in degrading the membrane‐bound insulin receptor. 39

This background on diabetes‐induced collagen molecular pathology resulted in the discovery experiments on the unexpected ability of tetracyclines to inhibit host‐derived matrix metalloproteinase activity by mechanisms unrelated to their antibiotic activity. The rationale for these experiments, and the resulting development of nonantibacterial tetracycline formulations and compositions, is reviewed below (for additional details, see references^1^, ^2^, ^13^, ^24^).

Moreover, the beneficial therapeutic results from a number of clinical trials in which the administration of the nonantimicrobial doxycycline‐based host‐modulation drug was tested as an adjunct to scaling and root planing have been reviewed over several decades, including the following reports: (1) the American Dental Association evidence‐based guidelines, which supported this adjunctive treatment as safe and effective, whereas other local (ie, sustained‐release antibiotics or antiseptics, as well as photodynamic and laser therapies) and systemic (azithromycin and ampicillin antibiotic) treatments were not recommended;^40^, ^41^ and (2) several meta‐analyses, which also supported the efficacy of subantimicrobial‐dose doxycycline as an adjunct to nonsurgical periodontal therapy.^14^, ^42^, ^43^ Moreover, it should also be mentioned that in an extensive, multicenter, 6‐month clinical trial of 180 patients, scaling and root planing combined with systemically administered subantimicrobial‐dose doxycycline (host‐modulation therapy) and a topically applied antimicrobial (Atridox^®^) resulted in a significantly greater clinical improvement compared with scaling and root planing alone.^44^

The discovery experiments, which ultimately resulted in the first (and still the only) government‐approved host‐modulation drug for periodontal disease, have been described in several additional published studies,^1^, ^5^, ^6^, ^10^ and the reader is referred to these and other articles for details. It should also be recognized that not only is nonantibiotic doxycycline (also known as subantimicrobial‐dose doxycycline) the only host‐modulation therapy available by prescription for the management of periodontitis (Periostat^®^), a modified once‐a‐day sustained‐release version of nonantibiotic doxycycline is also the only matrix metalloproteinase inhibitor drug available by prescription to dermatologists (Oracea^®^), although it and Periostat^®^ can be prescribed off‐label for other dental and medical clinical applications. Other experimental matrix metalloproteinase inhibitors, commonly based on zinc‐binding hydroxamic acid peptides, have been found to be potent inhibitors of matrix metalloproteinases, but have resulted in significant clinical adverse events, as it is now recognized that low levels of these neutral proteinases are required to maintain normal physiologic connective tissue turnover.^12^, ^23^, ^45^

Thus, with the early discovery of excessive matrix metalloproteinase activity and abnormal collagen turnover in the gingival tissues of rats with diabetes (and, later, in humans with diabetes), 2 competing hypotheses were proposed to explain the mechanisms of accelerated periodontal breakdown during this systemic disease and which resulted in drug discovery.^2^, ^5^

Hypothesis 1: Diabetes and hyperglycemia alter the oral and crevicular environment (eg, oxygen levels, pH), thus promoting the overgrowth of anaerobic gram‐negative bacteria. This pathogenic microflora could then result in elevated levels of lipopolysaccharide (endotoxin) in the gingival crevice in individuals with diabetes, which would induce pathologically excessive host‐derived expression of collagenase, and consequent breakdown of collagen, in the adjacent gingival tissues; or
Hypothesis 2: Diabetes increases gingival collagenase activity and pathologic collagen breakdown by mechanisms independent of any bacterial “shifts” and reflects, instead, a diabetes‐induced alteration in the host response.

To identify which of these 2 hypotheses was correct, the tetracycline antibiotic, minocycline, was administered to rats with diabetes to suppress the diabetes‐induced altered crevicular microflora (hypothesis 1), and was found to reduce substantially the excessive host‐derived collagenase activity (in the absence of any reduction of hyperglycemia). These observations initially appeared to support hypothesis 1. However, when the experiment was repeated using germ‐free rats, maintained in germ‐free environments, minocycline therapy again reduced the pathologically excessive gingival collagenase activity in the rats with diabetes back to normal rat levels. These observations eliminated hypothesis 1 and supported hypothesis 2. Moreover, these data, plus additional in vivo and in vitro (mechanistic) studies, clearly demonstrated that tetracyclines (minocycline, doxycycline, other tetracyclines) had a previously unrecognized ability to inhibit mammalian (host) tissue‐derived collagenases (and later, other matrix metalloproteinases), and by mechanisms independent of their antibiotic activity (outlined in Table 1).^2^, ^5^ These findings immediately drove the development of nonantimicrobial formulations (of which doxycycline was found to be the most potent matrix metalloproteinase inhibitor) and compositions (the chemically modified tetracyclines, such as chemically modified tetracycline‐3; discussed below) of tetracyclines that would not result in the side effect of antibiotic‐resistant bacteria and fungal overgrowth with long‐term administration, as prolonged treatment would be needed to modulate the host response.

### 2.1 Therapeutic rationale

Golub et al^2^ then designed 2 strategies of drug development to suppress connective tissue breakdown, including bone loss during periodontitis, and other dental and medical diseases, using nonantibacterial tetracyclines. Both strategies have recently been reviewed by this group.^13^, ^24^ In brief, the first strategy involved the custom formulation, then testing, of capsules containing standard levels of doxycycline (50 and 100 mg), which have antibiotic activity, as well as capsules containing lower levels of doxycycline (30, 20, and 10 mg), which are recognized to have non therapeutic antibiotic activity. In this program of design and testing, the 20 mg formulation in clinical studies was found to produce peak blood levels that were significantly lower than the >1.0 μg/mL needed for antibiotic activity. This low‐dose formulation yielded peak blood levels of 0.3‐0.6 μg/mL compared with peak blood levels for the antibiotic (100 mg) doxycycline dose of 2‐5 μg/mL. Unlike the antibiotic regimen, which is known to result in antibiotic‐resistant bacteria in a short period of time (eg, 2 weeks),^46^ the regimen of 20 mg twice a day (once every 12 hours) showed no evidence, in long‐term clinical trials, of antibiotic resistance in the oral microflora or in the microflora from the intestine, vagina, and skin.^8^, ^47^ Similarly, the sustained‐release once‐per‐day formulation (Oracea^®^) of nonantimicrobial‐dose doxycycline did not result in the emergence of antibiotic‐resistant bacterial strains^13^, ^24^, ^49^. More recently, a National Institutes of Health‐funded clinical trial on 128 postmenopausal women found that a 2‐year regimen of subantimicrobial‐dose doxycycline (20 mg twice a day) showed no difference compared with placebo capsules (vehicle only) in the emergence of an antibiotic‐resistant microflora^47^, ^50^, ^51^ and no difference in adverse events between the control and subantimicrobial‐dose doxycycline groups over this prolonged period of time (Table 2). However, consistent with medical applications of subantimicrobial‐dose doxycycline, the women on the 2‐year regimen of the Food and Drug Administration‐approved dental medication did show a statistically significant reduction in dermatologic adverse events (Table 2) as well as statistically significant improvements in periodontal disease and systemic biologic parameters (Table 3). Beneficial long‐term results were also seen in clinical trials on patients with rheumatoid arthritis,^52^ acne,^49^ and other medical conditions, as discussed later in this review.

**Table 2:** Adverse events in postmenopausal women (n = 126 subjects) given Periostat^®^ or placebo twice a day for a 2‐y duration^a^, ^b^

Adverse events	Study drug group count (% of patients)		
	Placebo	Subantimicrobial‐dose doxycycline	*P* value
Ache/pain	35 (55)	33 (52)	.7
Arthritis/inflammation	5 (8)	7 (11)	.5
Cancer	3 (5)	2 (3)	>.9
Cardiac: blood pressure, cholesterol, myocardial infarction	5 (8)	4 (6)	>.9
Cold/cough/respiratory	28 (44)	34 (53)	.3
Dermatologic	11 (17)	1 (2)	.002^a^
Gastrointestinal upset	14 (22)	15 (23)	.8
Hearing/vision	5 (8)	0 (0)	.06
Infection	22 (34)	14 (22)	.1
Injury	5 (8)	5 (8)	>.9
Minor surgery	7 (11)	6 (9)	.8
Oral events and lesions	3 (5)	3 (5)	>.9
Osteoporosis	0 (0)	3 (5)	.2
Psychological/sleep/neurological	6 (9)	8 (12)	.6
Other	3 (5)	7 (11)	.2

a Adapted from Payne et al.^66^

b See Table 3 for the efficacy of a 2‐y regimen of Periostat^®^ in postmenopausal women with periodontitis and (systemic) osteopenia.

**Table 3:** Efficacy of a 2‐yr regimen of subantimicrobial‐dose doxycycline in postmenopausal women with periodontitis and (systemic) osteopenia: a double‐blind placebo‐controlled randomized clinical trial^a^, ^b^

A. Clinical results Overall intent‐to‐treat analysis: subantimicrobial‐dose doxycycline reduced periodontal disease progression, based on pocket depth measurements over time, by 19%‐43% (the latter among individuals not on concomitant medications) Subgroup analyses: (a) subantimicrobial‐dose doxycycline reduced bleeding on probing by 30% in nonsmokers, (b) subantimicrobial‐dose doxycycline reduced bleeding on probing by 34% in protocol‐adherent individuals, (c) subantimicrobial‐dose doxycycline reduced the odds of more progressive periodontal disease, based on probing depth changes with time, by 43%
B. Radiographic/subtraction radiography and computer‐assisted densitometric image analysis Subantimicrobial‐dose doxycycline reduced progressive alveolar bone height loss by 36% (per‐protocol analysis), and by 29% in women> 5 yr postmenopause Subantimicrobial‐dose doxycycline reduced loss of alveolar bone density in sites with pocket depths ≥ 5 mm
C. Gingival crevicular fluid biomarkers Subantimicrobial‐dose doxycycline reduced gingival crevicular fluid collagenase activity by 22%, which reflected a 60% reduction in matrix metalloproteinase‐8, the most dominant of three collagenases in gingival crevicular fluid Subantimicrobial‐dose doxycycline reduced gingival crevicular fluid ICTP by 19% (collagenase activity and type I collagen carboxyterminal telopeptide were positively correlated at baseline, 1‐, and 2‐yr time periods) Subantimicrobial‐dose doxycycline reduced gingival crevicular fluid interleukin‐1beta by 51% in women > 5 yr postmenopause
D. Systemic benefits Subantimicrobial‐dose doxycycline significantly (*P* = .003) reduced serum ICTP and marginally reduced carboxy‐terminal collagen crosslinks (CT X)(*P* = .06) bone resorption biomarkers, with no effect on bone formation (the doxycycline blood level, 0.59 μg/mL, was consistent with the pharmacokinetics of subantimicrobial‐dose doxycycline therapy in humans) Subantimicrobial‐dose doxycycline reduced the serum inflammatory biomarkers, high sensitivity C‐reactive protein and matrix metalloproteinase‐9 Among women more than 5 yr postmenopause, subantimicrobial‐dose doxycycline increased the high‐density lipoprotein cholesterol level

a Modified from Payne and Golub^51^ and Golub et al.^54^, ^64^

b All results described in sections A, B, C, and D were statistically significant, *P* ≤ .05.ICTP, type I collagen carboxyterminal telopeptide.

## 3 CURRENT CLINICAL HOST‐MODULATION AGENTS: THERAPEUTIC EFFICACY AND SAFETY

As reviewed by our group and others,^1^, ^2^, ^11^, ^13^, ^14^ the repeated demonstration of tetracycline's unexpected ability, by nonantibiotic mechanisms, to inhibit pathologic collagenolysis (including bone loss) during periodontal diseases and other diseases, drove the development of 2 novel, systemically administered, therapeutic regimens: (a) nonantibiotic formulations of doxycycline, Food and Drug Administration‐approved for the treatment of periodontitis (Periostat^®^, now generic) and acne/rosacea (Oracea^®^) (both medications have also been approved in Canada as Periostat^®^ and Apprilon^®^ and in Europe as Periostat^®^ and Efracea^®^) and (b) a series of nonantibacterial compositions of tetracyclines. These novel compounds were created by structural modification (removal of the dimethylamino group at the carbon‐4 site of the A ring), which eliminated their antibacterial activity. However, they were also designed to retain and enhance the ability to inhibit matrix metalloproteinase activity, in part by preserving the zinc‐ and calcium‐binding beta‐diketone moiety at carbon‐11 and carbon‐12 of the tetracycline molecule. It should be noted that confirmation of this beta‐diketone as the active site for matrix metalloproteinase inhibition was achieved by structurally eliminating this cation‐binding moiety. This was accomplished by converting the tetracycline molecule to its pyrazole analog, which lacks this metal ion‐binding site, and, which, as a result, did not inhibit matrix metalloproteinases. One of the most potent chemically modified tetracyclines as a matrix metalloproteinase inhibitor, chemically modified tetracycline‐3, has been tested in phase I and phase II clinical trials by the National Cancer Institute and Harvard Medical School, and in much lower doses in preliminary studies on patients with chronic periodontitis.^25^, ^26^ The results of these studies are presented later in this review.

### 3.1 Safety and efficacy of subantimicrobial‐dose doxycycline as an adjunct to nonsurgical and surgical periodontal therapy in humans

As described earlier, a number of studies over the past several decades have demonstrated that scaling and root planing plus host‐modulation therapy, with subantimicrobial‐dose doxycycline as the adjunct, produced improved clinical results compared with scaling and root planing plus a placebo, as assessed by traditional diagnostic measures, including probing depth, clinical attachment level, bleeding on probing, radiographic bone loss,^2^, ^11^, ^44^, ^53^ and reduced biomarkers/mediators of inflammatory/collagenolytic disease, including cytokines and chemokines (interleukin‐1beta, tumor necrosis factor‐alpha, interleukin‐6, interleukin‐17, monocyte chemoattractant protein‐1),^22^, ^54^ matrix metalloproteinases (notably matrix metalloproteinase‐8 and matrix metalloproteinase‐9), pyridinoline bone type I collagen breakdown fragments, or type I collagen carboxyterminal telopeptide (ICTP) and, by indirect mechanisms of action, also inhibited elastase (serpinolytic) activity. This therapeutic regimen also reduced pathologically excessive levels of reactive oxygen species (eg, sodium hypochlorous acid) and nitric oxide in gingival crevicular fluid and in the adjacent gingival tissues.^1^, ^2^, ^54^, ^55^ These and other observations are summarized in Tables 1 and 3. In addition, Lee et al^55^ reported that a novel combination host‐modulation therapeutic regimen (subantimicrobial‐dose doxycycline plus low‐dose flurbiprofen) enhanced the efficacy of subantimicrobial‐dose doxycycline in suppressing matrix metalloproteinases, in addition to reducing serpinolytic (alpha1‐antitrypsin‐degrading) activity, as a result of the ability of the nonsteroidal anti‐inflammatory drug to enhance the delivery of the matrix metalloproteinase inhibitor drug to the inflamed tissues, including the diseased gingiva and the arthritic joint.^55^, ^56^, ^57^

Of particular interest regarding the clinical efficacy of nonantimicrobial tetracyclines (especially doxycycline, a more potent matrix metalloproteinase inhibitor than tetracycline itself, or minocycline) as host modulators, is the issue of substantivity, which reflects the ability of the medication to produce a therapeutic effect for some time after drug administration has stopped.

In early studies, Golub et al^6^ administered subantimicrobial‐dose doxycycline to patients with periodontitis for 2 weeks (control subjects received placebo capsules twice a day) and reported “that this regimen dramatically reduced the mammalian collagenase activity, not only in the gingival crevicular fluid, but also in the adjacent gingival tissues that were surgically excised for therapeutic purposes.” However, subsequent clinical trials indicated that although increasing the regimen of subantimicrobial‐dose doxycycline to 4 weeks also reduced periodontitis biomarkers, cessation of host‐modulation therapy resulted in a rapid rebound of collagenase activity back to placebo levels.^10^, ^13^ As a result, the duration of host‐modulation therapy as an adjunct to scaling and root planing was increased in later clinical trials by various groups^10^, ^50^, ^53^, ^58^, ^59^ to regimens of 3, 6, and 9 months, and 1 and 2 years. This strategy provided solid evidence not only of prolonged clinical efficacy and safety, but also of substantivity, which is discussed below.

After early discovery studies demonstrated that short‐term regimens of subantimicrobial‐dose doxycycline inhibited inflammatory/collagenolytic biomechanisms of periodontal disease in humans, and without the emergence of antibiotic‐resistant microorganisms,^2^ longer‐term studies were then carried out.^8^ As an example of the impressive scope of some of these studies, Ciancio and Ashley^61^ described a multicenter, placebo‐controlled double‐blind randomized clinical trial of 531 patients with chronic periodontitis to assess long‐term efficacy of subantimicrobial‐dose doxycycline. In brief, clinical improvements were seen, over a 12‐month time period, in pocket depth, clinical attachment level, bleeding on probing, alveolar bone height (assessed using subtraction radiography), and disease activity (defined as “incidences of rapid progression”).^61^ Moreover, the 1‐year administration of subantimicrobial‐dose doxycycline produced a safety profile of adverse events that was the same as for the placebo (there was also no effect on diagnostic laboratory parameters, including liver and kidney function), and no microbial resistance was developed to tetracyclines or other antibiotics in the patients treated with subantimicrobial‐dose doxycycline.

Subsequent clinical studies addressed the substantivity of host‐modulation therapy, namely the ability of subantimicrobial‐dose doxycycline to produce therapeutic benefits for months after the patient has stopped taking the medication, which are now discussed.

Although Caton and Ryan^10^ reported that a 1‐month treatment with subantimicrobial‐dose doxycycline was not sufficient to produce a long‐term benefit, a 3‐month regimen did produce prolonged improvement during the subsequent no‐treatment phase of the study. Consistent with these findings, a 1‐year double‐blind placebo‐controlled study of 190 participants with chronic periodontitis, which involved 5 dental centers, reported significant reductions in probing depth, gains in clinical attachment, and prevention of disease progression, over a 9‐month time period of subantimicrobial‐dose doxycycline therapy. These clinical benefits were maintained for at least 3 months after the treatment was stopped.

This substantivity of clinical improvement was also seen in studies of patients with rapidly progressing severe periodontitis. In one such study, participants with severe disease were administered a 6‐month regimen of subantimicrobial‐dose doxycycline adjunctive to repeated scaling and root planing treatments. The dramatic improvement in clinical response, compared with repeated scaling and root planing plus placebo treatments, was maintained for at least 3 months after the cessation of host‐modulation therapy.^14^, ^48^, ^62^ In another study, a 3‐month regimen of subantimicrobial‐dose doxycycline adjunctive to nonsurgical therapy administered to smokers with severe periodontitis also produced significant substantivity.^53^ Moreover, a demonstration of prolonged substantivity was provided by Emingil et al.^63^ In this 1‐year placebo‐controlled double‐blind study, the patients with periodontitis were administered adjunctive subantimicrobial‐dose doxycycline for only the first 3 months. Of note, the improved clinical measures (gingival index, probing depth, clinical attachment gain) and biomarker responses (gingival crevicular fluid levels of interleukin‐6, tumor necrosis factor‐alpha, monocyte chemoattractant protein‐1), compared with placebo administration, were maintained for at least 9 months after the cessation of medication.

A more recent 2‐year randomized double‐blind placebo‐controlled study of 128 postmenopausal women, a category of individuals at risk for progressive periodontal disease and bone loss, provides further evidence for the safety and efficacy of prolonged subantimicrobial‐dose doxycycline therapy. These data are summarized in Tables 2 and 3.^50^, ^51^, ^54^, ^64^, ^65^, ^66^ As shown in Table 2, there was no evidence of adverse events in the postmenopausal women taking subantimicrobial‐dose doxycycline, adjunctive to nonsurgical therapy, for the 2‐year study period compared with placebo.^66^ However, 1 category did show a significant effect, but it cannot be described as an adverse event; namely, the women who were administered subantimicrobial‐dose doxycycline showed a highly significant (*P* = .002) 90% reduction in dermatologic adverse events. As discussed below, this observation supports the efficacy of subantimicrobial‐dose doxycycline in managing a variety of chronic inflammatory/collagenolytic diseases, such as (but not limited to) rheumatoid arthritis, cardiovascular and pulmonary diseases, as well as dermatologic diseases, including acne and rosacea. Regarding periodontitis in the postmenopausal women in the above study, the 2‐year regimen of subantimicrobial‐dose doxycycline produced significant improvements (compared with placebo administration) in clinical measures (pocket depth, bleeding on probing, and periodontal disease progression), radiographic bone loss (observed using subtraction radiography and computer‐assisted densitometric image analysis), and gingival crevicular fluid biomarkers (cytokines, matrix metalloproteinases, and ICTP; Table 3). Also of interest, particularly in this group of individuals who exhibited mild systemic (skeletal) bone loss diagnosed as osteopenia, the postmenopausal women who were administered subantimicrobial‐dose doxycycline showed a significant reduction in ICTP in their systemic circulation, indicating that this host‐modulation therapy for their periodontal disease might also be reducing their risk for developing more severe systemic bone loss or osteoporosis.^50^, ^64^

Additional evidence of efficacy, safety, and substantivity of subantimicrobial‐dose doxycycline, as adjunctive therapy following periodontal surgery, was provided by the Forsyth/Michigan group.^9^ In this 1‐year study, 24 patients with severe chronic periodontitis were administered subantimicrobial‐dose doxycycline (Periostat^®^) or placebo capsules twice a day for the first 6 months after access flap surgery. The response to this host‐modulation therapy was assessed by clinical measures, gingival crevicular fluid bone‐resorption biomarkers (ICTP), and microbial DNA analysis of multiple bacterial species. The most profound clinical outcome of subantimicrobial‐dose doxycycline therapy compared with placebo was a significant reduction of pocket depth, a benefit that was still observed 6 months after discontinuing this host‐modulation therapy. The authors concluded that subantimicrobial‐dose doxycycline promoted more rapid wound healing “by preventing collagen disruption and indirectly encouraging collagen formation,” interpretations consistent with earlier observations listed in Table 1 on the ability of nonantibiotic tetracyclines to upregulate collagen synthesis, osteoblast activity, and bone formation.

In summary, numerous short‐ and long‐term clinical trials, over several decades of investigation, have demonstrated, without any contradictory evidence, that subantimicrobial‐dose doxycycline (Periostat^®^), now generic in the USA, and more recently Oracea^®^, are safe and effective adjuncts in the management of chronic periodontitis and its more aggressive forms. Regarding these more aggressive forms of periodontitis^147^, subantimicrobial‐dose doxycycline has also demonstrated efficacy in reducing periodontal disease severity in highly susceptible categories of patients, including institutionalized geriatric individuals,^67^ smokers,^14^, ^53^ patients with diabetes,^68^, ^69^ and cardiovascular patients.^70^, ^71^, ^72^ Later sections will address the therapeutic potential of chemically modified tetracyclines and of the dosage regimens of nonantibiotic tetracyclines in the management of several medical diseases, including the fields of ophthalmology, dermatology, cardiac and pulmonary disorders, diabetes, arthritis, and cancer.

## 4 THE CHEMICALLY MODIFIED TETRACYCLINES: DEVELOPMENT AND PRELIMINARY CLINICAL STUDIES

Soon after the development of nonantibiotic formulations or dosage regimens of doxycycline (20 mg twice a day) as safe and effective host‐modulator medications, a second strategy emerged involving the chemical modification of the tetracycline (or doxycycline or minocycline) molecule to eliminate its antibacterial activity selectively, but to retain or even enhance its matrix metalloproteinase‐inhibitory (host‐modulating) properties. As first described by Golub et al,^2^, ^147^ this involved the removal of a side‐chain on the tetracycline molecule, the dimethylamino group at carbon‐4 of the A ring, which is responsible for the drug's antibiotic activity, and the preservation of the zinc‐ and calcium‐binding site (the beta‐diketone moiety) at carbon‐11 and carbon‐12, which we identified as the site on the tetracycline molecule responsible for its matrix metalloproteinase‐inhibitory activity. The elimination of this metal ion‐binding site, by chemically converting the tetracycline molecule to its pyrazole analog, eliminated the drug's matrix metalloproteinase‐inhibitory properties.^2^ As a result of these, and additional chemical modifications, a series of nonantibacterial matrix metalloproteinase‐inhibitory tetracyclines was developed. One of these, chemically modified tetracycline‐3, was found, based on in vitro and animal studies, to be a potent matrix metalloproteinase inhibitor with significant therapeutic potential.^24^ In vitro, chemically modified tetracycline‐3 was found to inhibit matrix metalloproteinase expression and activity in human colon and breast cancer cells, in addition to cytostatic effects on human renal and prostate cancer. In vivo administration of this novel compound to rats with prostate cancer inhibited tumor growth and metastasis.^2^, ^73^, ^74^, ^75^, ^76^ As a result of these and other studies, the National Cancer Institute (National Institutes of Health) in collaboration with Dezube et al 26 (Beth Israel Deaconess Hospital, Harvard Medical School) tested chemically modified tetracycline‐3 in phase I and phase II clinical trials. In phase I trials, the maximum tolerable dose was determined to be 150 mg/d. When administered orally, the compound was found to be rapidly absorbed, producing a peak blood level of 2‐5 μg/mL, with a prolonged serum half‐life of approximately 40 hours.^75^, ^77^, ^78^ In this study, chemically modified tetracycline‐3 (CMT‐3, or COL‐3) was administered once a day to patients with AIDS‐related Kaposi sarcoma, who exhibited a 44% response rate, reflecting a decrease of angiogenic lesions, which was associated with a suppression of matrix metalloproteinase‐2 levels in the circulation. In a larger phase II clinical trial, a group of patients with Kaposi sarcoma, who received a daily oral dose of 50 mg of chemically modified tetracycline‐3, showed a significant reduction of angiogenic lesions, whereas the individuals who were administered the higher dose (100 mg once a day) did not. One explanation is that the increased side effects of the higher dose resulted in decreased patient compliance. Significant reductions in plasma levels of matrix metalloproteinase‐2 and matrix metalloproteinase‐9 were observed, and the most common adverse events were photosensitivity and rash, which could be quite severe.^26^, ^75^

The same compound was found to reduce mortality in a pig and other animal models of an often‐fatal lung disorder, acute respiratory distress syndrome, and showed evidence of efficacy in models of septic shock.^79^, ^80^, ^81^, ^82^ The only human study testing chemically modified tetracycline‐3 on periodontal disease was a pilot trial, conducted by Ryan et al.^25^ A low dose of chemically modified tetracycline‐3 (10 mg/d) was administered to patients with chronic periodontitis and showed modest evidence of efficacy in reducing interleukin‐1beta and matrix metalloproteinase‐8 in gingival crevicular fluid samples, with no evidence of the severe side effect of photosensitivity previously seen in the extensive studies of Kaposi sarcoma patients using higher doses of chemically modified tetracycline‐3. As described below, newer chemically modified polyphenolic compounds derived from a common food supplement, curcumin, are being developed by the Stony Brook group. These compounds are potent inhibitors of matrix metalloproteinases and reduce the levels of inflammatory mediators. They appear to be much safer than chemically modified tetracycline‐3, although clinical trials on humans have not yet been carried out.

## 5 NONANTIBIOTIC TETRACYCLINES: ADDITIONAL CLINICAL APPLICATIONS IN MEDICINE

Because collagen is the major structural protein in connective tissues, including (but not limited to) skin, bone, tendon, ligaments, cornea of the eye, cartilage of the joints, etc., and because the only host‐derived neutral proteinases able to degrade the triple‐helical collagen molecule under physiologic conditions of pH (7.4‐7.6) and body temperature are the collagenolytic matrix metalloproteinases (essentially the three collagenases matrix metalloproteinase‐1, matrix metalloproteinase‐8, and matrix metalloproteinase‐13, and, to a lesser extent, matrix metalloproteinase‐2 and matrix metalloproteinase‐14), it is not surprising that any new medication that can inhibit these matrix metalloproteinases would be expected to have widespread medical as well as dental applications. In this regard, two special issues of the journal, *Pharmacological Research*, had “Clinical Applications of Non‐antibacterial Tetracyclines” as their theme and most chapters focused on medical disorders including cardiovascular and pulmonary diseases, dermatology, ophthalmology, rheumatology, and cancer, with extensive detail on each disease.^33^, ^34^

### 5.1 Ophthalmology

**Figure 1:**
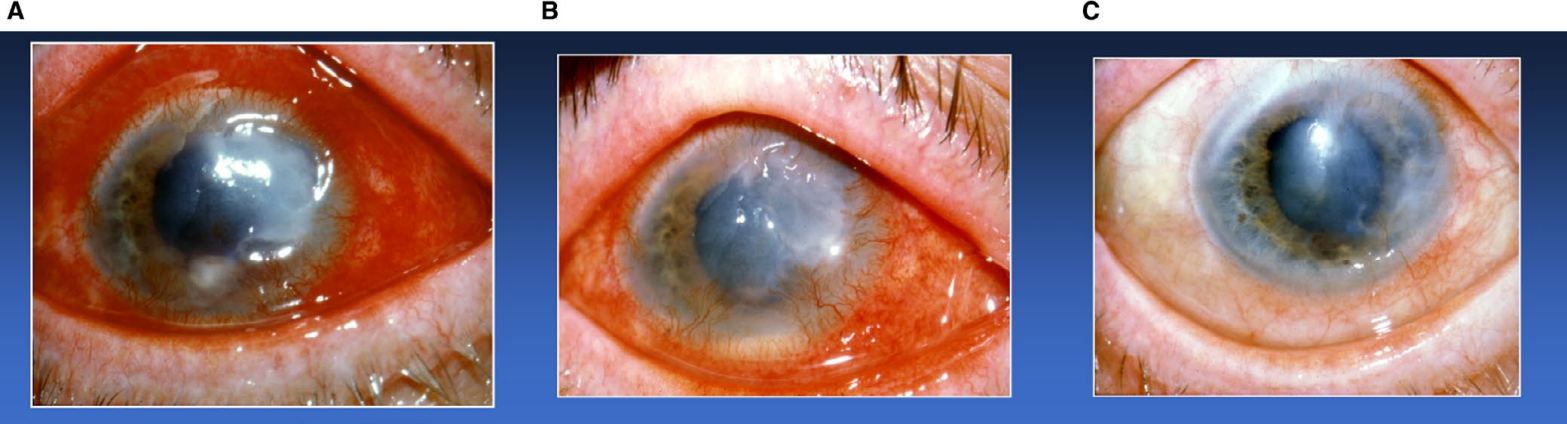
The clinical response of a patient with a sterile corneal ulcer refractory to standard ophthalmological treatment. A, Note the “moon crater” painful ulcer associated with severe inflammation of the sclera. B, Significant healing of the sterile corneal ulcer and reduction of inflammation 48 hours after initiating doxycycline anticollagenase therapy. C, Complete healing after 1 month of doxycycline administration (reproduced with permission from Henry Perry, MD)

Soon after the discovery of tetracyclines as matrix metalloproteinase inhibitors, one of the first medical applications was for noninfected or sterile corneal ulcers.^83^ Perry reported that a patient with a sterile corneal ulcer, refractory to standard treatment, responded dramatically within 48 hours after initiating tetracycline therapy, with no recurrence long term (see Figure 1). In a follow‐up case series involving 18 patients with persistent sterile corneal defects, most patients had healed lesions within 48 hours after initiating oral tetracycline therapy and all lesions were resolved within 2 weeks.^84^ Given that various cell types in the cornea, including the epithelium, fibroblasts, macrophages, and neutrophils, produce a variety of matrix metalloproteinases (matrix metalloproteinase‐1, ‐8, ‐2, ‐9), the response to tetracycline host‐modulatory therapy, although profound, was not surprising.^85^ Subsequently, both topical and systemic tetracycline therapies were effective in rabbit models of sterile corneal ulcers^86^ and all three tetracyclines, including minocycline and doxycycline, were effective inhibitors of corneal collagenases.^87^ Federici^88^ reviewed a number of studies using tetracyclines for sterile corneal ulcers, chemical burns of the eye, and blepharitis of the eyelid, and concluded that these (and other ophthalmic diseases, such as cataract, macular degeneration, and diabetic retinopathy) may benefit from the nonantibiotic properties of tetracyclines and other novel matrix metalloproteinase inhibitors.

### 5.2 Dermatology

Early reports on tetracycline's efficacy in serious dermatologic lesions, soon after their matrix metalloproteinase‐inhibitory properties were discovered,^1^, ^2^ focused on the hope that this safe collagenase inhibitor would be useful in a rare, and often fatal, skin disease, dystrophic epidermolysis bullosa. In this regard, White^89^ described two cases of dystrophic epidermolysis bullosa in which minocycline therapy substantially reduced dermal blisters and ulcers. The rationale for this therapy was the demonstration, in patients with epidermolysis bullosa, of excessive collagenase (matrix metalloproteinase‐1) and other matrix metalloproteinases (matrix metalloproteinase‐3), which could accelerate degradation of type VII collagen anchoring the dermis to the type IV collagen of the epidermal basement membrane.^90^, ^91^ However, in a more recent review, Monk et al^49^ did not find sufficient evidence to support treatment of epidermolysis bullosa with tetracyclines. The same authors, however, did conclude that tetracyclines, by their nonantibiotic properties, were efficacious in a more common skin blistering disorder, bullous pemphigoid. The mechanism proposed involved tetracycline's inhibition of various matrix metalloproteinases (matrix metalloproteinase‐2, matrix metalloproteinase‐9, matrix metalloproteinase‐13), which mediate the degradation of a basement membrane constituent, type XVII collagen. Another dermatologic application was described by Humbert et al.^90^ They found that doxycycline treatment completely healed the sterile, deep, painful skin ulcers in patients with alpha1‐antitrypsin‐deficiency panniculitis. This disorder affects a significant number of northern Europeans characterized by a genetic deficiency of this endogenous proteinase inhibitor, which can also be degraded by matrix metalloproteinases. Thus, doxycycline, by directly inhibiting matrix metalloproteinases (including their ability to degrade the serum protein, alpha1‐antitrypsin) enables even any remaining low levels of this endogenous serine proteinase inhibitor in these patients to continue preventing the breakdown of dermal connective tissue constituents.

Finally, Cohn et al^92^ reported that patients with benign mucus membrane pemphigoid showed a reduction of oral mucosal blisters and ulcers after a 3‐month regimen of nonantibiotic doxycycline. In this oral disease, nonantibiotic doxycycline inhibited accelerated degradation of the hemidesmosomal protein, type XVII collagen, by matrix metalloproteinase‐9, thus preventing blister formation. Anecdotal observations indicated enhanced efficacy when this regimen was combined with a nonsteroidal anti‐inflammatory drug, consistent with similar observations in rheumatology (discussed below).

However, the most immediate medical impact of nonantibiotic doxycycline has been on the common skin disorders, acne and rosacea. As described earlier in this chapter, a major clinical trial on postmenopausal women that focused on the long‐term response of periodontal and systemic (osteopenia) bone loss to nonantimicrobial doxycycline resulted in a 90% (*P* = .002) reduction in dermatologic adverse events (eg, acne, rash) compared with the incidence of adverse events in individuals given a placebo (Table 2).

As summarized by Monk et al,^49^ rosacea is characterized by erythema patches in the facial skin, as well as other inflammatory lesions, including pustules and papules, and swollen veins (telangiectasia) in the nose and cheeks. The repeated success of subantimicrobial‐ or nonantimicrobial‐dose doxycycline, either in a novel sustained‐release formulation of 40 mg once per day (ie., Oracea^®^) (note: 40 or 50 mg doxycycline, as standard formulations, do produce antibiotic blood levels and side effects) or as now‐generic, 20 mg twice a day (Periostat^®^), "provides the most abundant clinical evidence, to date, that tetracyclines are effective through their nonantimicrobial actions."^49^ The efficacy of subantimicrobial‐dose doxycycline in these patients has been ascribed to multiple mechanisms of action, including decreased cytokine production, reduced inducible nitric oxide synthase activity, reactive oxygen species inhibition, antiangiogenesis, and collagen preservation as a result of both matrix metalloproteinase inhibition and enhanced collagen production.^49^ The literature supporting the safety and efficacy of subantimicrobial‐dose doxycycline in this dermatologic disorder is substantial. Among the most extensive published clinical studies, Del Rosso et al^93^ carried out 2 multicenter, 4‐month phase III clinical trials of 537 adults and found significant improvements in numbers of inflammatory skin lesions (papules, pustules, nodules) and decreased erythema. In a subsequent study, the same investigators^94^ found that although nonantibiotic doxycycline was as efficacious as higher (100 mg) antibiotic‐dose doxycycline, the lower dose produced significantly fewer adverse events. Subantimicrobial‐dose doxycycline was also found to be significantly more effective in treating rosacea, even when combined with topical metronidazole.^95^ As with rosacea, acne has also responded significantly to subantimicrobial‐dose doxycycline in randomized clinical trials, with reductions in inflammatory skin lesions, including papules, pustules, and comedones. Nonantibacterial mechanisms of action of doxycycline and other tetracyclines have been ascribed to the inhibition of interleukin‐8, lipoxygenase, matrix metalloproteinase‐1, and reactive oxygen species, as well as altered neutrophil chemotaxis and activation.^49^, ^96^, ^97^

### 5.3 Rheumatology

As reviewed by Greenwald,^57^ early studies on the scientific basis for tetracycline treatment of arthritic disorders were focused on the drug's antibiotic properties and, later, on its anti‐inflammatory efficacy, the latter characterized by reduced leukocyte chemotactic and phagocytic activity. Then, in a seminal paper, Greenwald et al^56^ described data on a cohort of patients with arthritis who required bilateral total knee replacements. In each of 6 patients with rheumatoid arthritis, the synovial tissue from one knee was harvested, with the tissue from the other knee being harvested 2 weeks later. Minocycline or placebo capsules were administered orally on a daily basis during the interval. When the tissues were extracted, partially purified, and analyzed in a blinded protocol, the inflamed synovial tissues of the patients treated with minocycline showed a 67% reduction in matrix metalloproteinase/collagenase activity compared with the tissues of patients given a placebo. Smith et al^98^ followed a similar protocol in patients with osteoarthritis, with similar results obtained using doxycycline.^98^ Israel et al,^99^ in a dentally relevant case study, described clinical and radiologic evidence of improvement in osteoarthritis of the temporomandibular joint. Perhaps the most compelling evidence of tetracycline's host‐modulatory efficacy in arthritis was the long‐term clinical trial by O'Dell et al.^52^ Sixty‐six patients diagnosed with rheumatoid arthritis, based on clinical, radiographic, and biomarker (rheumatoid factor‐positive) criteria, were administered subantimicrobial‐dose doxycycline (20 mg), 100 mg of doxycycline, or placebo capsules over a 2‐year period. All patients received standard‐of‐care methotrexate, in addition to the test medications. Both groups that were given doxycycline exhibited a similar 2‐ to 3‐fold improvement in rheumatoid arthritis scores (eg, joint pain, number of swollen joints, grip strength, erythrocyte sedimentation rate, etc.) compared with the control subjects, and the subantimicrobial‐dose doxycycline group experienced the same incidence of adverse events as the group that were given a placebo. By contrast, the individuals given higher antibiotic‐dose doxycycline showed more adverse events over the 2‐year protocol.

Animal studies (rats, dogs) on experimentally induced rheumatoid arthritis and osteoarthritis also provide further evidence of the efficacy of nonantibiotic tetracyclines as host modulators. In some of these studies, a chemically modified tetracycline (eg, chemically modified tetracycline‐1; 4‐dedimethylamino tetracycline) was tested and, when combined with a nonsteroidal anti‐inflammatory drug (such as indomethacin, flurbiprofen), a synergistic therapeutic response was observed.^100^, ^101^ Subsequent studies demonstrated that the administration of doxycycline in human osteoarthritis produced these benefits in association with suppression of the matrix metalloproteinases, collagenase and gelatinase.^98^

### 5.4 Cardiovascular, pulmonary, and other medical disorders

The research and development of nonantibiotic tetracyclines as host modulators continues to expand into additional medical conditions. However, because these initial studies and biologic mechanisms have been reviewed extensively,^13^, ^33^, ^34^ and because the results of longer‐term randomized clinical trials that demonstrate the efficacy of host‐modulation therapies on diseases such as cardiovascular disease, pulmonary disorders, etc., have yet to be reported, these topics will only be briefly discussed.

Regarding cardiovascular disease, animal studies have shown reductions in the severity of hypertension and aortic aneurysm expansion, ascribed in part to inhibition of matrix metalloproteinase‐9 and matrix metalloproteinase‐12 by chemically modified tetracycline and doxycycline, which preserves collagen and elastic fibers in blood vessel walls.^72^, ^102^, ^103^ Regarding atherosclerosis, the analysis of thousands of records by Meier et al^104^ identified a reduced risk for acute myocardial infarction in those patients who had been treated for infection with a tetracycline over a 5‐year period. By contrast, no effect was seen in those patients treated with a different antibiotic (erythromycin, penicillin, cephalosporin). Initially, the antibiotic superiority of tetracyclines to suppress *Chlamydia pneumoniae* (previously, but not currently, associated with cardiovascular disease) was speculated. However, Golub et al^71^ proposed that tetracycline's unique ability to inhibit matrix metalloproteinase‐mediated rupture of the collagen‐coated atheromatous plaque lining coronary arteries was the mechanism. A study showing evidence of the efficacy of subantimicrobial‐dose doxycycline in patients with acute coronary syndromes^70^ provides support for this hypothesis.

Several short‐term (1 and 6 months) double‐blind placebo‐controlled studies of patients with cardiovascular disease have shown promising responses on biomarkers of systemic inflammation and collagenolysis. Brown et al^70^ reported that patients with acute coronary syndrome who were administered Periostat^®^ for 6 months showed significant reductions in high‐sensitivity C‐reactive protein, interleukin‐6, and matrix metalloproteinase‐9 in their plasma. In addition, elevations in cardioprotective high‐density lipoprotein cholesterol (and its core molecule, ApoA1 lipoprotein) were also seen in patients exhibiting both cardiovascular disease and periodontitis who were treated with scaling and root planing and adjunctive Periostat^®^.^105^, ^106^

Similar beneficial cardiovascular disease biomarker responses in a long‐term (2‐year) Periostat^®^ study were confirmed in a major National Institutes of Health‐supported randomized placebo‐controlled clinical trial of 128 postmenopausal women, a patient cohort at risk for cardiovascular disease.^51^, ^65^ Schulz^107^ provided a novel mechanism for the observations described above, which involved the previously unrecognized ability of tetracyclines to inhibit matrix metalloproteinase degradation of intracellular contractile proteins (actin, myosin, troponin) in the cytoplasm of cardiac myocytes.

In pulmonary disease, the most dramatic life‐saving response to the host‐modulating properties of nonantibiotic doxycycline was that described by Moses et al^108^ in a patient with lymphangioleiomeiomatosis. Her case report in the *New England Journal of Medicine* was soon followed‐up with similar positive results in 30 patients in Brazil.^109^ In brief, this rare and fatal lung disease is characterized by the progressive loss of lung function mediated by uncontrolled invasion of lung tissue by neoplastic smooth muscle cells, which express excessive levels of matrix metalloproteinases, especially the collagen and elastic fiber‐degrading gelatinases/type IV collagenases, matrix metalloproteinase‐2 and matrix metalloproteinase‐9. Treatment was initiated with subantimicrobial‐dose doxycycline (20 mg once a day, then 20 mg twice a day), which began to reduce symptoms and biomarkers. The dose was then increased to 100 mg, with significant clinical improvement. The result was an improved quality of life and the cancellation of lung transplant surgery. Another pulmonary disease that has responded, at least in animal models, including a clinically applicable porcine model, is acute respiratory distress syndrome.^82^ In this, and other studies, a potent matrix metalloproteinase‐inhibitor, nonantimicrobial chemically modified tetracycline‐3, reduced signs and symptoms, including fatal outcomes.^81^, ^82^

### 5.5 Diabetes and postmenopausal bone loss

The impact of nonantibiotic tetracycline host modulators (chemically modified tetracyclines and subantimicrobial‐dose doxycycline) on two major medical conditions, namely diabetes and postmenopausal bone loss (oral and skeletal), has been summarized in several articles, including a recent review by our group.^24^

Historically, the concept of host‐modulation therapy originated from early experiments by our group targeting the impact of type 1 diabetes on periodontal inflammation, collagen turnover, and bone loss.^2^, ^4^, ^5^, ^6^ The breakthrough occurred when tetracycline therapy was found to modulate the host response effectively even in germ‐free rats with diabetes. Subsequent studies by Ryan et al^68^ found similar periodontal and systemic biologic changes in rats with type 2 diabetes treated with host‐modulating tetracyclines. One long‐held controversy in this field involved the reported efficacy of nonsurgical periodontal therapy (scaling and root planing combined with oral hygiene instruction and antiseptic mouthrinses) in reducing hyperglycemia in patients with diabetes.^110^, ^111^ As recently reviewed,^24^ this was subsequently challenged in a multi‐institutional clinical trial by Engebretson et al,^112^ who did not find any significant improvement in glycated hemoglobin levels as a result of nonsurgical periodontal therapy. The same author reported that although scaling and root planing alone, or combined with adjunctive antibiotic therapy, did not significantly reduce glycated hemoglobin levels, when a 3‐month regimen of host‐modulation therapy (nonantibiotic doxycycline, 20 mg twice a day) was added to the scaling and root planing treatment protocol, the level of glycated hemoglobin was reduced.^58^ Clearly this important topic in dentistry and medicine needs further investigation to confirm whether subantimicrobial‐dose doxycycline, with prolonged administration, can significantly reduce hyperglycemia; and whether host‐modulating periodontal therapy should be incorporated into the management protocol for diabetes, which is an increasingly prevalent and devastating disease worldwide.

The final topic to be discussed, before addressing newer host‐modulation therapies being developed (see Section 6), is the local and systemic benefits of subantimicrobial‐dose doxycycline in postmenopausal women with periodontitis (local bone loss) and skeletal (systemic) osteopenia.^51^ The substantial basic science foundation for launching this major National Institute of Dental and Craniofacial Research National Institutes of Health‐supported and ‐monitored long‐term study included the following early observations: tetracyclines, by nonantibacterial mechanisms, were found to inhibit, directly, excessive bone resorption in organ culture,^113^ osteoclast activity in cell culture,^114^ and alveolar bone loss in experimental periodontitis.^1^, ^2^, ^116^ The biologic rationale for these studies included the following: type 1 collagen constitutes 90% of the organic matrix of bone, and matrix metalloproteinases (notably the collagenases and gelatinases, and others, such as membrane type 1‐matrix metalloproteinase/matrix metalloproteinase‐14) produced by bone cells (osteoclasts, osteoblasts, osteocytes) mediate collagenolysis during bone resorption. Thus, the matrix metalloproteinase‐inhibitory properties of tetracyclines and their chemically modified nonantimicrobial analogs (the chemically modified tetracyclines) could be expected to downregulate bone resorption. Unexpectedly, however, these host‐modulatory agents were also found to upregulate bone formation. Evidence for this pro‐anabolic tetracycline effect included host‐modulation therapy‐enhanced expression of type 1 procollagen mRNA, together with increased collagen synthesis and bone formation, all of which were suppressed in rats with diabetes.^114^, ^116^, ^117^, ^118^ Another compelling rationale for testing subantimicrobial‐dose doxycycline in a randomized clinical trial of postmenopausal women was an early study by Golub et al.^119^ Using the standard animal model of postmenopausal osteoporosis, the ovariectomized rat, the investigators found that the oral administration of chemically modified nonantibiotic doxycycline (chemically modified tetracycline‐8) inhibited gingival matrix metalloproteinase activity and periodontal breakdown, while also reducing the severity of skeletal osteoporosis. Consistent with these observations, Scarpellini et al^120^ reported that doxycycline administered to postmenopausal women “normalized” biomarkers of bone formation (osteocalcin and alkaline phosphatase) and bone resorption (urinary excretion of hydroxyproline).

A preliminary 1‐year clinical trial was then carried out on postmenopausal women who were diagnosed with both local (periodontitis) and systemic (osteopenia/osteoporosis) bone loss. Subantimicrobial‐dose doxycycline, compared with treatment with placebo capsules, as an adjunct to periodontal maintenance therapy, reduced alveolar bone loss and suppressed the progressive loss of clinical attachment.^50^ The totality of the in vitro, animal, and preliminary clinical study just described, led to a major bi‐institutional (University of Nebraska and Stony Brook University) double‐blind clinical trial on 128 postmenopausal women administered subantimicrobial‐dose doxycycline or placebo capsules for 2 years adjunctive to periodontal maintenance therapy every 3‐4 months. These data were published in a variety of dental, medical, and biology journals, and were summarized by Payne and Golub.^51^ In brief, the 2‐year regimen of host‐modulation therapy did not produce adverse events, only benefits, (ie, the women on Periostat showed a 90% [*P* = .002] reduction in dermatologic adverse events, which included acne, rosacea, and rash) (Table 2). Regarding examples of efficacy in this extensive study, a 36% lower odds of progressive alveolar bone loss was found in women within 5 years of menopause, for those who were protocol‐adherent, compared with those administered placebo (*P* = .03), plus significant reductions in both clinical attachment loss and active pockets. The postmenopausal women on subantimicrobial‐dose doxycycline also showed significant reductions in collagenase, predominantly matrix metalloproteinase‐8, in gingival crevicular fluid, as well as significant reductions in bone collagen degradation fragments (ICTP) in periodontal pockets (Table 3). A similar response of ICTP and a newer biomarker of bone collagen degradation (carboxy‐terminal collagen crosslinks) in serum samples led to the conclusion that this host‐modulation therapy reduced the risk of mild systemic bone loss, osteopenia, progressing into the more severe form of skeletal deficiency, osteoporosis.^64^

## 6 FUTURE HOST‐MODULATION AGENTS: THE CHEMICALLY MODIFIED CURCUMINS

### 6.1 Introduction

**Figure 2:**
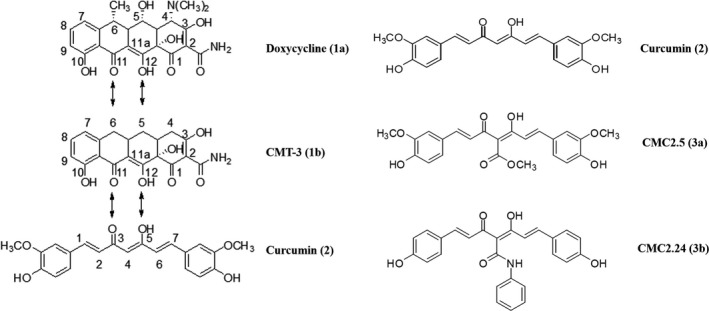
The development scheme of first‐generation (1a, nonantibiotic doxycycline formulations), second‐generation (1b, nonantibiotic chemically modified tetracycline‐3 [CMT‐3]), and third‐generation (3a, b, chemically modified curcumins [CMCs]) host‐modulating therapeutics. This strategy was based on these tetra‐, tri‐, and diphenolic compounds all exhibiting the Zn^++^‐binding beta‐diketone (1a, 1b, 2) or triketone (3a, 3b) moieties in the structures illustrated^30^, ^31^

Although the discovery of the unexpected property of tetracyclines as matrix metalloproteinase inhibitors has been translated into novel first‐generation (Periostat^®^, Oracea^®^) and second‐generation (chemically modified tetracycline‐3) pharmaceutical agents, a third generation of related host‐modulating therapeutics is now being developed (Figure 2). Based on our previous identification of the matrix metalloproteinase‐inhibiting active site on the 4‐phenolic‐ring tetracyclines,^1^, ^2^, ^24^, ^147^ we began to focus on diphenolic compounds with the same cation (Zn^++^, Ca^++^)‐binding site, the beta‐diketone moiety. These included the bis‐aroyl methanes and curcumin. The bis‐aroyl methane compounds exhibited positive, but weak, matrix metalloproteinase‐inhibitory efficacy in vitro, namely high IC_50_ (μm) values, and were quickly abandoned.^30^, ^31^ Curcumin, a dietary herbal ingredient derived from turmeric, has historically been advocated as a safe and effective treatment for a variety of diseases.^30^, ^31^, ^32^ However, it has long been known that this compound's insolubility and poor absorption by the oral route has limited its clinical application.^123^, ^124^, ^125^

Recently, our Stony Brook University laboratories (Oral Biology and Chemistry departments, Dr. Francis Johnson) have synthesized, developed, and tested a series of novel chemically modified curcumins with various side chains added to the carbon‐4 position of this biphenolic compound (Figure 2). Based on these studies, a lead triphenolic compound, a triketonic phenylaminocarbonyl curcumin‐2.24, was identified, which has been tested in vitro, in cell and tissue culture, and in vivo using various animal species, including mice, rats, rabbits, and, most recently, dogs.

### 6.2 Discovery and therapeutic rationale

Based on their in vitro efficacy as inhibitors of matrix metalloproteinase‐mediated degradation of a fluorogenic synthetic peptide substrate with the susceptible glycine‐leucine peptide bond, the original series of 16 different chemically modified curcumins, all with a carbon‐4 substituent, was reduced to several promising novel triketonic compounds. Two of these, chemically modified curcumin‐2.24 (4‐phenylaminocarbonyl curcumin) and chemically modified curcumin‐2.5 (4‐methoxycarbonyl curcumin), were then compared with diketonic natural curcumin to determine their potency as matrix metalloproteinase inhibitors. Chemically modified curcumin‐2.24 was found to bind most strongly to Zn^++^ and to serum albumin, consistent with its enhanced potential to function in vivo (discussed below) as a potent inhibitor of Zn^++^‐dependent collagenases, and to retard decomposition of the serum albumin‐bound chemically modified curcumin, thus enhancing its bioavailability.^30^, ^31^ Preliminary cell culture studies on human monocytes stimulated with microbial lipopolysaccharide/endotoxin demonstrated that chemically modified curcumin‐2.24 was superior to chemically modified curcumin‐2.5 as an inhibitor of matrix metalloproteinase‐9 (92 kDa gelatinase). A similar result was seen in macrophages derived from rats with diabetes that were orally administered the same compounds.^30^, ^31^ Therefore, although chemically modified curcumin‐2.5 was superior to the parent compound curcumin as a matrix metalloproteinase‐9 and a matrix metalloproteinase‐13 (collagenase‐3) inhibitor in vitro, with similar effects in vivo, it has since been abandoned in favor of chemically modified curcumin‐2.24. Like chemically modified curcumin‐2.5, curcumin‐2.24 was also more effective than curcumin in suppressing the inflammatory mediators interleukin‐1beta, interleukin‐6, prostaglandin E_2_, and monocyte chemoattractant protein‐1, as well as matrix metalloproteinase‐9 secretion, by human monocytes in culture. The effects of chemically modified curcumin‐2.24 were recently associated with decreased activation/phosphorylation of nuclear factor kappa‐light‐chain‐enhancer of activated B cells, which regulates transcription of a number of gene products associated with inflammatory diseases.^29^

In addition to chemically modified curcumin‐2.24 exhibiting superior efficacy compared to curcumin or chemically modified curcumin‐2.5 as an inhibitor of inflammatory mediators and matrix metalloproteinases, this novel compound was found to be even more effective in reducing alveolar bone loss in vivo. In this regard, Guimarães et al^126^ reported that although curcumin was effective in suppressing inflammatory mediators in the rat model of lipopolysaccharide‐induced periodontal disease, natural curcumin had no effect on alveolar bone loss, an observation that was later confirmed.^127^ They also found that natural curcumin and chemically modified curcumin‐2.24 reduce the severity of inflammation in experimental periodontal disease by different mechanisms, and only the latter decreased the number of osteoclasts characterized immunohistochemically as tartrate‐resistant acid phosphatase‐positive (TRAP‐positive) and caspase‐3‐positive cells. Elburki et al,^27^, ^28^ in a preliminary study (later confirmed; see below) using the same rat model of periodontitis, found that pathologic alveolar bone loss was completely prevented by orally administered chemically modified curcumin‐2.24. This dramatic effect on periodontal bone loss in vivo was associated with suppression of the proinflammatory cytokine, interleukin‐1beta, and reduced levels of pro and activated forms of leukocyte‐type gelatinase (matrix metalloproteinase‐9) in the gingival tissues. Moreover, this locally induced periodontitis, and its treatment with chemically modified curcumin‐2.24, exhibited systemic manifestations ie, the elevated levels in the circulation of interleukin‐1beta and the activated form of matrix metalloproteinase‐8 [leukocyte‐type collagenase] in the circulation of rats with experimental periodontitis were both significantly reduced by oral administration of chemically modified curcumin.

To explore further the potential of our lead compound as a host modulator, chemically modified curcumin‐2.24 was orally administered to 2 different animal models of severe periodontal breakdown 27, 28, 29. In both the locally (microbial endotoxin/lipopolysaccharide) and the systemically (diabetes)‐induced rat models of disease, chemically modified curcumin‐2.24 treatment resulted in a marked reduction of inflammatory cytokines and matrix metalloproteinases in the gingival tissues, decreased bone loss, and decreased activation of p65 nuclear factor kappa‐light‐chain‐enhancer of activated B cells and p38 mitogen‐activated protein kinase pathways.^29^ These data further supported the potent ability of this chemically modified curcumin to modulate intra‐ and extracellular mechanisms that are associated, at least in part, with the zinc‐binding characteristics of this and related compounds.^29^ This pattern of efficacy is also consistent with the pleiotropic characteristics of the zinc‐binding nonantimicrobial tetracyclines.^2^, ^24^

The potency of chemically modified curcumin‐2.24 therapy has also been demonstrated in a challenging, but clinically relevant, dental/medical situation using an animal model that mimics a patient with poorly controlled diabetes and severe periodontitis. Once again, chemically modified curcumin‐2.24 did not reduce the severe hyperglycemia (at least during the 1‐month experimental protocol), yet completely prevented alveolar bone loss, which was induced by multiple lipopolysaccharide injections into the gingiva of rats with severe diabetes.^29^ Addressing the causality of this decreased periodontal breakdown, this novel therapeutic also suppressed the excessive levels of pro‐ and activated matrix metalloproteinase‐2 and matrix metalloproteinase‐9 (gelatinases/type IV collagenases) in the diseased gingival tissues, as well as the activated (lower molecular weight) form of the dominant collagenase, matrix metalloproteinase‐8, in these oral tissues. The dominant cytokines in the inflamed gingiva, interleukin‐1beta and interleukin‐6 (tumor necrosis factor‐alpha was not detected), were also reduced locally by this treatment, and similar beneficial biomarker changes were seen systemically in the circulation, as well as in skin. Collagen atrophy and excessive collagen crosslinking (ie, “leather‐like” texture) were also normalized in the animals with diabetes that were treated with chemically modified curcumin‐2.24. This is of particular relevance to a common medical complication in patients with diabetes ie, impaired healing of wounds in skin, gingiva, and other tissues 32.

The clinical complications (adverse events) associated with the severely hyperglycemic state in this rat model of type 1 diabetes, which included bleeding under the nails, inflammation of the sclera, and impaired wound healing, were also prevented by this treatment.^28^, ^29^ It should be noted that impaired wound healing in skin, especially in the lower extremities, can be a life‐threatening complication in patients with diabetes and is a major cause of limb amputation in humans.^128^, ^129^ This important complication and other serious medical disorders, and their response to chemically modified curcumin‐2.24, are also discussed in the next section.

### 6.3 Chemically modified curcumin: additional medical implications

#### 6.3.1 Wound healing

The novel host‐modulation therapy highlighted in this review was recently found to be exceptionally effective in normalizing, not just improving, the healing of skin wounds categorized as standard and severe in rats with type 1 diabetes.^32^ Of particular interest, the therapeutic response occurred even in the presence of severe hyperglycemia (blood glucose levels of 500 mg/dL) and was equally effective when applied either topically or systemically. A 1% suspension of chemically modified curcumin‐2.24, applied topically, produced optimal results (assessed using clinical, biochemical and histomorphometric measurements) based on a significant reduction of pathologically excessive matrix metalloproteinase‐8 (collagenase‐2) and hydroxyproline levels in wound tissue. An additional mechanism currently being investigated is the ability of chemically modified curcumin‐2.24 to normalize a key resolvin (RvD1; a docosahexanoic acid derivative) and interleukin‐10 (an anti‐inflammatory cytokine), which are both underexpressed in macrophages isolated from severely hyperglycemic rats with diabetes.^130^, ^131^

#### 6.3.2 Pulmonary disease

A recent study by Wang et al^122^ demonstrated that chemically modified curcumin‐2.24, administered by oral gavage, was more effective than natural curcumin in reducing lung injury in a standard mouse model of pneumonia and acute respiratory distress syndrome. In brief, *Staphylococcus aureus* administered by intratracheal inoculation was used to create the lung disease. Based on analysis of bronchioalveolar lavage fluid and lung tissue, chemically modified curcumin‐2.24 significantly reduced lung tissue apoptosis. In bronchoalveolar lavage fluid, alveolar neutrophils and macrophages, the matrix metalloproteinases, matrix metalloproteinase‐2, matrix metalloproteinase‐9 (gelatinases, type IV collagenases), matrix metalloproteinase‐12 (macrophage metallo‐elastase), and nuclear factor kappa‐light‐chain‐enhancer of activated B cells 65 were all significantly reduced. The authors concluded that, in this model, chemically modified curcumin has the potential to attenuate lung injury and reduce mortality.

#### 6.3.3 Arthritis

In contrast to the tetracycline‐based host modulators that have been studied extensively in human and experimental rheumatoid arthritis and osteoarthritis, the chemically modified curcumins are just beginning to receive attention. In this regard, Katzap et al^132^ compared the efficacy of several of these novel compounds, including chemically modified curcumin‐2.14, ‐2.5 and ‐2.23, and our lead compound, chemically modified curcumin‐2.24, with natural curcumin, in a standard tissue culture model of arthritis. Bovine cartilage explants, labeled with S^35^‐sulfate, were incubated with interleukin‐1beta and oncostatin to induce cartilage breakdown. Chemically modified curcumin‐2.24 was the compound most effective in reducing chondrolysis. In vivo studies using the rabbit model of arthritis are currently underway to confirm the therapeutic potential of this compound.

#### 6.3.4 Cancer

Taking into account the previous studies described in this paper on the chemically modified tetracyclines in humans and animals with various cancers, and the similar cation‐binding site of curcumin and these nonantibacterial tetracycline compounds, several studies using chemically modified curcumin‐2.24 are now discussed. As a new generation taxol, SBT‐1214, was found to downregulate the expression of multiple genes in cancer stem cells in culture, and as natural curcumin was found to enhance the cytotoxic effects of a number of anticancer drugs, Botchkina et al^133^ tested this taxoid in combination with the novel curcuminoid, chemically modified curcumin‐2.24. They found that this treatment was more effective than SBT‐1214 alone in upregulating proapoptotic genes and suppressing multiple transcription factors in prostate cancer stem cells. As stated by the authors, this experimental therapeutic regimen has “high potential as a novel anti‐cancer drug combination.”^133^ Moreover, the potent characteristics of chemically modified curcumin‐2.24 as an inhibitor of matrix metalloproteinases is consistent with these host‐derived proteinases functioning as stromal effectors of carcinoma progression.^134^

As this application of chemically modified curcumin in cancer treatment continues to emerge, several recent studies support this novel therapeutic approach. Wright et al^121^ reported that in a mouse model of medulloblastoma, treatment with chemically modified curcumin‐2.24 combined with a curcumin‐phosphatidyl choline (to improve bioavailability) showed therapeutic efficacy in controlling tumor growth. Of particular interest, Mackenzie's group at Stony Brook's Cancer Center recently demonstrated that chemically modified curcumin‐2.24 induced apoptosis in pancreatic cancer cells and reduced pancreatic tumor growth by inhibiting active Ras and signal transducer and activator of transcription‐3 phosphorylation.^135^ Moreover, even at high (1000 mg/kg) oral doses, the animals treated with chemically modified curcumin‐2.24 showed no evidence of toxicity (Heta Bhatt et al., unpublished data).

## 7 FUTURE HOST‐MODULATION CANDIDATES FOR CLINICAL APPLICATION

Several additional host modulators have also demonstrated potential therapeutic value based on in vitro studies and animal studies but have not yet reached the stage of development to justify randomized clinical trials. Further studies on animal models of periodontal disease are also required. These compounds include the sirtuins and resveratrol.

As reviewed recently by Mendes et al,^136^ the subset of sirtuins that are of particular relevance to periodontal disease include 7 proteins (sirtuins 1‐7) with different subcellular locations, mitochondria, cytoplasm, or nucleus. However, because of their deacylation activity, the nuclear sirtuins (sirtuins 1, 2, 6, and 7) in particular have been the focus of efficacy studies in several inflammatory diseases. One example of this strategy includes sirtuin 1, which suppresses nuclear factor kappa‐light‐chain‐enhancer of activated B cells activity, thus decreasing the expression of inflammatory mediators, cyclooxygenase‐2, inducible nitric oxide synthase, and the cytokines, interleukin‐1beta, tumor necrosis factor‐alpha, interleukin‐6, and interleukin‐8.^137^

Resveratrol, the phytochemical component of red wine often credited for its multiple health benefits, activates sirtuin 1^138^ and increases brown adipose tissue as part of complex cellular processes associated with aging, apoptosis, and inflammation.^139^ sirtuin 1 and other sirtuins can be inhibited by Zn^++^‐binding agents, such as the matrix metalloproteinase inhibitor, hydroxamic acid, suggesting additional mechanisms and therapeutic medications already highlighted in this review, including the nonantibiotic tetracyclines and curcuminoids. Although clinical trials testing these futuristic host modulators, (eg, resveratrol) for various diseases, such as diabetes, cancer, psoriasis, and colitis, are ongoing, their potential role in periodontitis has only been addressed in a relatively small number of animal studies. However, a recent study by Ikeda et al,^140^ using a mouse model of ligature‐induced periodontitis, provides grounds for optimism. Using techniques of morphometry/microcomputed tomography to quantify alveolar bone loss 3‐dimensionally, quantitative reverse transcriptase‐PCR to assess gene expression of gingival tissue cytokines (interleukin‐1beta, interleukin‐6), as well as quantifying tartrase‐resistant acid phosphatase‐positive osteoclasts in cell culture, the authors found that a natural source of resveratrol inhibited periodontal breakdown associated with decreased oxidative stress and reduced osteoclast differentiation and activity. Regarding resveratrol's therapeutic potential, significant issues remain, including poor bioavailability associated, in part, with rapid urinary excretion.^141^

## 8 CONCLUDING COMMENTS AND FUTURE DIRECTIONS

With the increasing recognition of the overarching importance of the host's inflammatory/collagenolytic responses as the "driver" of tissue breakdown during periodontitis, new treatment paradigms are emerging. Accordingly, this chapter has highlighted the 2 most‐developed host‐modulation therapy strategies to date. However, the specialty of periodontics has increasingly incorporated the placement of dental implants in its treatment strategies. This reflects, at least in part, its (and the public's) recognition of the significant enhancement of the dentition's function and esthetics by this technology.

However, a surprisingly high incidence of peri‐implant mucositis and peri‐implantitis (19%‐65%) has been recognized as a significant complication, but with very limited treatment options (see the editorial by Giannobile and Lang^142^). Considering the well‐established negative impact of periodontitis around natural teeth, on systemic inflammation and health (eg, increased risk for cardiovascular disease), similar adverse systemic effects may also occur with peri‐implant diseases. This chapter has highlighted the safety and efficacy of government‐approved host‐modulation therapies for periodontal disease and their systemic benefits. There is now a compelling rationale for future studies: (1) that define the impact, if any, of peri‐implant disease on systemic inflammation; and (2) that assess the efficacy of host‐modulation therapies both locally (on inflamed peri‐implant tissues) and systemically (on circulating biomarkers of "inflammatory/collagenolytic" diseases) as adjuncts to conventional local disinfection treatments. These proposals are also driven by several studies that have addressed new diagnostic concepts. In this regard, Xu et al^143^ demonstrated a higher level of leukocyte‐type collagenase (matrix metalloproteinase‐8) in peri‐implantitis sulcular fluid than that seen in gingival crevicular fluid from patients with periodontitis. Moreover, based on numerous clinical studies (as recently reviewed by Alassiri et al^144^ and Al‐Majid et al^145^), Sorsa et al 144, 145have translated into clinical practice a novel chairside diagnostic test that detects pathologically elevated levels of activated matrix metalloproteinase‐8 in both gingival crevicular fluid and peri‐implantitis sulcular fluid. In closing, these novel diagnostic and therapeutic developments provide a "window" into future clinical applications of the basic and translational research described in this review.

## DISCLOSURES

Drs Golub and Lee are listed as inventors on patents on the medications/compounds described in this review, and these have been fully assigned to their institution, Stony Brook University, State University of New York.
